# Differentiation of Human Induced Pluripotent Stem Cell (hiPSC)-Derived Neurons in Mouse Hippocampal Slice Cultures

**DOI:** 10.3389/fncel.2017.00143

**Published:** 2017-05-17

**Authors:** Toshimitsu Hiragi, Megumi Andoh, Toshihiro Araki, Takayuki Shirakawa, Takashi Ono, Ryuta Koyama, Yuji Ikegaya

**Affiliations:** ^1^Laboratory of Chemical Pharmacology, Graduate School of Pharmaceutical Sciences, University of TokyoTokyo, Japan; ^2^Advanced Drug Research Laboratories, Mitsubishi Tanabe Pharma CorporationYokohama, Japan

**Keywords:** iPS cells, neural differentiation, transplantation, hippocampus, slice culture

## Abstract

Potential clinical applications of neurons derived from human induced pluripotent stem cells (hiPSC-neurons) for drug screening and transplantation therapies have received considerable attention. However, it remains unclear whether and how transplanted hiPSC-neurons are incorporated into pre-existing neural circuits. Here we developed a co-culture system of hiPSC-neurons and mouse hippocampal slices to examine the differentiation of hiPSC-neurons in pre-existing neural circuits. hiPSC-neurons transplanted in mouse hippocampal slices expressed the hippocampal neuron-specific markers HuB and Prox1 after 7 days of culture, while those markers were scarcely expressed in hiPSC-neurons cultured on glass dishes. Furthermore, hiPSC-neurons transplanted in the dentate gyrus (DG) of slice cultures grew to exhibit dentate granule cell-like morphologies, including besom-shaped dendrites. Similarly, hiPSC-neurons transplanted in the CA1 region of slice cultures grew to exhibit CA1 pyramidal cell-like morphologies, including primary apical and multiple basal dendrites with synaptic spines. Additionally, these cells projected axons toward the entorhinal cortex (EC) as observed *in vivo*. These data suggest that hiPSC-neurons were anatomically integrated into pre-existing neural circuits in a region-specific manner. Thus, the co-culture system will be useful for the study of efficient strategies to differentiate transplanted hiPSC-neurons.

## Introduction

Neurons derived from pluripotent stem cells such as embryonic stem cells (ESCs) or induced pluripotent stem cells (iPSCs) have received considerable attention for clinical applications (Goldman, 2005). Among the clinical applications, stem cell-based transplantation therapy has been anticipated in the field of neuroscience for the replacement of injured or dead neurons in patients with neurodegenerative diseases. Since neurons are not reproduced throughout life except in limited brain regions, stem cell-based transplantation therapy is an important approach to compensate for neuronal loss. Previous studies that used mouse or human ESC/iPSC-derived neurons have shown that stem cell transplantation is useful for various types of diseases. For example, in animal models of Parkinson’s disease, motor coordination was restored with the transplantation of human ESC/iPSC-derived dopaminergic neurons to the striatum (Roy et al., 2006; Hargus et al., 2010). In animal models of spinal cord injury, motor function was recovered after the transplantation of human iPSC-derived neural stem cells to injured areas (Nori et al., 2011). Additionally, in animal models of stroke, the transplantation of human iPSC-derived long-term expandable neuroepithelial-like stem cells into the striatum improved sensorimotor recovery (Oki et al., 2012; Tornero et al., 2013), and in animal models of temporal lobe epilepsy, seizures were attenuated by transplanting ESC-derived GABAergic interneurons to the hippocampus (Cunningham et al., 2014).

ESC/iPSC-derived neurons could acquire specific neuronal subtypes (Imaizumi et al., 2015; Sakaguchi et al., 2015) and form functional synapses in dissociated cultures on glass dishes (Kim et al., 2011; Shi et al., 2012) or *in vivo* (Espuny-Camacho et al., 2013). Furthermore, it has been shown that transplanted neural stem cells (Englund et al., 2002) or embryonic neurons (Falkner et al., 2016) exhibit electrophysiological properties of host neurons and are functionally integrated into pre-existing neural circuits *in vivo*. However, considering future clinical applications, it is difficult to use embryonic neurons or ESC-derived neurons because of ethical issues and biological difficulties mainly caused by immune responses in recipients. To overcome these critical issues, it is essential to use human iPSC derived-neurons (hiPSC-neurons) and to develop methods to differentiate iPSC-neurons in the transplanted milieu. Specifically, transplanted hiPSC-neurons are required to exhibit brain region-specific morphologies because the highly polarized characteristic morphologies of each neuronal subtype are the basis for the mediation of brain region-specific neural transmission (Larkman and Mason, 1990; Mason and Larkman, 1990; Mainen and Sejnowski, 1996; Krichmar et al., 2002). However, to date, a convenient experimental system with which to examine whether and how transplanted hiPSC-neurons develop brain-region specific morphologies is lacking.

Here we developed a co-culture system of hiPSC-neurons and mouse hippocampal slices. We found that hiPSC-neurons transplanted and cultured in hippocampal slices grow to express neuronal subtype-specific markers, while hiPSC-neurons cultured on a glass dish fail to express those makers. Furthermore, hiPSC-neurons transplanted in the dentate granule cell layer (GCL) or CA1 pyramidal cell layer (PCL) exhibited granule cell-like or pyramidal cell-like morphologies, respectively. Additionally, immunohistochemical analysis revealed that the transplanted hiPSC-neurons form dendritic spines that are colocalized with synaptic markers. In summary, hiPSC-neurons could develop brain region-specific morphologies in our co-culture system, and the experimental system may be useful for the future screening of factors that are necessary for the differentiation of transplanted hiPSC-neurons.

## Materials and Methods

### Animals and Ethics

C57BL/6J male mice (SLC, Shizuoka, Japan) and Thy1-membrane-targeted GFP (mGFP) transgenic mice (Lsi1; gifts from Dr. Caroni) were housed in cages in standard laboratory conditions with a 12-h light/dark cycle and free access to food and water. All experimental procedures conformed to the National Institutes of Health Guide for the Care and Use of Laboratory Animals and animal experiments were performed with the approval of the animal experiment ethics committee at the University of Tokyo (approval number: 24-70) and according to the University of Tokyo’s guidelines for the care and use of laboratory animals. All efforts were made to minimize the animals’ suffering and the number of animals used. For the preparation of primary hippocampal cultures and hippocampal slice cultures, mouse pups were decapitated after being deeply anesthetized on ice, and their mothers were euthanized with isoflurane.

### Maintenance of Human iPSCs

Human iPSCs were cultured in the single-cell and feeder-free (SFF) culture system as previously described (Ono et al., 2014). Briefly, cells were grown at 37°C and 5% CO_2_ in MT-fCFA medium, a modified chemically defined medium (MT-CDM) supplemented with activin A (338-AC; R&D Systems, Minneapolis, MN, USA) and FGF2 (100-18B; Peprotech, Rocky Hill, NJ, USA). For passaging, the cells were treated with 0.005% trypsin/0.002% EDTA (Sigma-Aldrich, St. Louis, MO, USA) for 3 min, mixed with 250 μg/ml of trypsin inhibitor (Thermo Fisher Scientific, Waltham, MA, USA), centrifuged at 180× g for 5 min, and resuspended in MT-fCFA medium containing 2.4 μM thiazovivin (Wako, Osaka, Japan) and 4.7 μg/ml human fibronectin (FN; 356008; Corning, NY, USA). The cells were plated on collagen type I (Col I)-coated dishes (IWAKI, Tokyo, Japan) at a dilution ratio of 1:5. Cells routinely received fresh medium every day and were passaged when 70%–80% confluence was reached, which normally occurred every 2–3 days. For cryopreservation, cells were suspended in STEM-CELLBANKER (BLC-3S; ZENOAQ, Fukushima, Japan) and frozen at −80°C, following the manufacturer’s instructions.

### *In Vitro* Neuronal Differentiation

Neuroectoderm (NE) induction was achieved by dual-smad inhibition (Chambers et al., 2009) for 7 days in the MT-CDM medium with 10 μM SB431542 (SB; Wako) and 0.3 μM LDN193189 (LDN; Stemgent, Lexington, MA, USA). At day 7, the resulting cells were dissociated with Accutase (Merck Millipore, Darmstadt, Germany) and transferred to suspension culture dishes (Sumitomo Bakelite, Shinagawa, Tokyo) in the MT-fCF medium (MT-fCFA medium without activin A) containing 10 μM SB. The cells were cultured for 7 days and allowed to form neurosphere-like spheroids. Then, the spheroids were dissociated with Accutase and plated back onto poly-L-ornithine (PLO)/laminin-coated dishes for 14 days in Neurobasal medium (Thermo Fisher Scientific) supplemented with B-27 Supplement Minus Vitamin A (12587-010; Thermo Fisher Scientific) and 1 μM cyclopamine (LKT laboratories, St. Paul, MA, USA) to promote the generation of cortical progenitor-like cells. The medium was changed every 2–3 days in all culture steps. The differentiated cells were cryopreserved at −80°C in a STEM-CELLBANKER.

### Dissociated Culture of Human iPSC-Derived Neurons

Cryopreserved hiPSC-neurons were thawed with thawing medium, which consisted of Neurobasal medium (Thermo Fisher Scientific) supplemented with B-27 Supplement Minus Vitamin A (Thermo Fisher Scientific) (1:50), N-2 supplement (17502-048; Thermo Fisher Scientific) (1:100), GlutaMAX supplement (35050-061; Thermo Fisher Scientific) (1:100) and Penicillin (10,000 units/ml)/Streptomycin (10,000 μg/ml) (1:100). Thawed cell solutions were centrifuged at 1000 rpm for 5 min, and the supernatant was removed. Cells were dissolved and plated on poly-D-lysine coated 12 mm glass coverslips (Neuvitro Corp., Vancouver, WA, USA) at a density of 1~5 × 10^4^ cells/well with culture medium; thawing medium with 4.71 μg/mL FN (Corning), 20 ng/mL BDNF (450-02; Peprotech), 20 ng/mL GDNF (450-10; Peprotech), 100 μM dcAMP (N6,2′-O-Dibutyryladenosine 3′,5′-cyclic monophosphate sodium salt; D0260; Sigma-Aldrich), 64.5 μg/mL L-ascorbic acid 2-phosphate trisodium salt (323-44822; Wako), 100 ng/mL IGF-1 (291-G1; R&D Systems) and 20 ng/mL NT-3 (267-N3; R&D Systems) was added. Half of culture medium was replaced with culture medium without FN (Corning) twice weekly.

### Primary Hippocampal Culture

Hippocampal cultures were prepared from postnatal day 1 (P1) C57BL/6J mice. The hippocampus was dissected in warmed (37°C) Hank’s balanced salt solution (HBSS), minced, and incubated at 37°C for 15 min with trypsin/EDTA (Sigma-Aldrich) followed by incubation at room temperature for 5 min with DNase (Sigma-Aldrich). Tissue was washed with HBSS three times. HBSS was replaced with Neurobasal plating medium (Neurobasal Medium containing B27 Supplement (1:50), 0.5 mM Glutamine Solution, 25 μM Glutamate, Penicillin/Streptomycin (1:200), 1 mM HEPES, 10% horse serum (26050-088; HS, heat-inactivated and filter-sterilized, Gibco, Grand Island, NY, USA). Tissue was triturated with a fire-polished Pasteur pipette and filtered through a 40-μm-pore cell strainer (Corning). Neurons were plated on poly-D-lysine coated 12 mm glass coverslips at a density of 6 × 10^4^ cells/well, and placed in a 37°C, 5% CO_2_ incubator. At 1 day, *in vitro* (1 DIV) neurobasal plating medium was replaced with neurobasal feeding medium (Neurobasal medium containing B27 Supplement (1:50), 0.5 mM Glutamine Solution, Penicillin/Streptomycin (1:200), 1 mM HEPES). At 2 DIV, cytosine arabinoside (AraC; Sigma-Aldrich) was added to a final concentration of 5 μM to inhibit the proliferation of dividing non-neuronal cells, and the medium was replaced with fresh neurobasal feeding medium 24 h after AraC was added. After 3 DIV, half of the neurobasal feeding medium was replaced with fresh neurobasal feeding medium every 4 days.

### Organotypic Culture of Hippocampal Slices

Mouse hippocampal slice cultures were prepared as previously described (Koyama et al., 2007; Kasahara et al., 2016) from P6 C57BL/6J mice or P10 Thy1-mGFP mice. Briefly, the posterior part of the mouse brain was cut into 400-μm thick transverse slices with a DTK-1500 vibratome (Dosaka, Kyoto, Japan) in aerated, ice-cold Gey’s balanced salt solution (GBSS) containing 36 mM glucose. The slices were incubated for 30–90 min at 4°C in incubation medium containing minimal essential medium (MEM) and HBSS at a ratio of 2:1, 9.0 mM Tris, 22.9 mM HEPES, and 63.1 mM glucose supplied with penicillin/streptomycin. Following this incubation, the slices were placed on Omnipore® membrane filters (JHWP02500; Merck Millipore) on doughnut plates (Hazai-Ya, Tokyo, Japan) in a solution containing 50% MEM, 25% horse serum, 25% HBSS, 6.6 mM Tris, 16.9 mM HEPES and 4.0 mM NaHCO_3_ supplemented with 29.8 mM glucose and 1% gentamicin sulfate solution (16672-04; Nacalai Tesque, Kyoto, Japan). Finally, the slices were cultured at 35°C in a humidified incubator with 5% CO_2_ and 95% air. The culture medium was changed twice weekly.

### Transplantation of hiPSC-Neurons to Slice Cultures

Ten microlitres of hiPSC-neuron solutions (1.0~5.0 × 10^5^ cells/mL) were dropped onto hippocampal slice cultures at 4 DIV. The hiPSC-neurons were additionally cultured on cultured hippocampal slices for 7 or 14 days.

### *In Vitro* Electroporation

hiPSC-neurons in thawing medium (see “Dissociated Culture of hiPSC-Derived Neurons” Section) were certificated at 1000 rpm at room temperature for 5 min, and the supernatant was removed. hiPSC-neurons were diluted in Opti-MEM (31985-062; Thermo Fisher Scientific) and subsequently electroporated with plasmids (10 μg plasmids in 100 μL cell solution) with NEPA21 Super Electroporator (poring pulse: pulse voltage, 275 V; pulse length, 1 ms; pulse interval, 50 ms; number of pulses, 2; decay rate, 10%; NEPAGENE, Chiba, Japan) equipped with a CU500 chamber for a cuvette electrode, a CU600 rack for a cuvette electrode, and an entorhinal cortex (EC)-002S NEPA cuvette electrode set (NEPAGENE). Within 1 min after electroporation, cells were cultured on a glass dish or cultured hippocampal slices. For visualizing the morphologies of hiPSC-neurons, the membrane-targeted *Aequorea coerulescens* green fluorescent protein (AcGFP)-coding plasmid, pAcGFP1-Mem was purchased from Takara Bio (Shiga, Japan) and used.

### Immunocytochemistry and Immuno-histochemistry

The following primary antibodies were used: mouse anti-Human nuclei (1:200; Merck Millipore), rabbit anti-Cleaved Caspase-3 (1:500; Cell Signaling Technology, MA, USA), rabbit anti-HuB (1:500; Abcam, Cambridge, UK), rabbit anti-prox 1 (1:500; Synaptic Systems, Göttingen, Germany), rabbit anti-Homer1 (1:500; Synaptic Systems), guinea pig anti-vGlut1 (1:500; Synaptic Systems), mouse anti-MAP2 (1:1000; Merck Millipore), rabbit anti-glial fibrillary acidic protein (GFAP; 1:500; Synaptic Systems), and chicken anti-GFP (1:1000; Abcam). Secondary antibodies conjugated with Alexa fluor dyes (1:500; Thermo Fisher Scientific) were used. For labeling the cell body of neurons, the NeuroTrace® 435/455 Blue Fluorescent Nissl Stain (1:200; Thermo Fisher Scientific) was used.

Immunocytochemistry for hiPSC-neurons and hippocampal neurons was carried out as follows. The cultured hiPSC-neurons and hippocampal neurons on a glass dish were fixed with 4% PFA at 37°C for 30 min. The samples were rinsed 5 min × 3 times with PBS and permeabilized for 1 h at 4°C in 0.1 M phosphate buffer and 0.1% Triton X-100 with 5% goat serum (Vector Laboratories, Burlingame, CA, USA). The samples were subsequently incubated with the primary antibodies in 0.1 M phosphate buffer and 0.1% Triton X-100 with 5% goat serum overnight at 4°C. After they were rinsed with PBS for 10 min × 3 times, the samples were incubated with the secondary antibodies in 0.1 M phosphate buffer and 0.1% Triton X-100 with 5% goat serum for 6 h at 4°C. After they were rinsed three times for 10 min each with PBS, the samples were mounted with vectashield containing DAPI (Funakoshi, Tokyo, Japan).

Immunohistochemistry for cultured hippocampal slices was carried out as follows. The samples were fixed with 4% PFA at 4°C overnight. The fixed slices were rinsed for 10 min × 3 times with PBS. Next, the slices were permeabilized for 1 h at room temperature in 0.1 M phosphate buffer and 0.3% Triton X-100 with 10% goat serum under agitation. The samples were subsequently incubated with the primary antibodies in 0.1 M phosphate buffer and 0.3% Triton X-100 with 10% goat serum overnight at room temperature under agitation. After they were rinsed with PBS for 5 min × 3 times, the samples were incubated with the secondary antibodies in 0.1 M phosphate buffer with 10% goat serum overnight at room temperature under agitation. After they were rinsed with PBS for 10 min × 3 times, the samples were embedded in Permafluor (Thermo Fisher Scientific, Waltham, MA, USA).

### Image Analysis and Statistical Analysis

Images were acquired with an FV1200 scanning confocal microscope (Olympus, Tokyo, Japan) equipped with diode lasers (405, 473, 559 and 635 nm) and analyzed with ImageJ (NIH, Bethesda, MD, USA). For the images of whole slice cultures, Z-series images were acquired with a 0.40 NA 10× objective at a voxel size of 1.242-1.242-4.51 μm (x-y-z). For the analysis of colocalization of the Human Nuclei and HuB or Prox1, Z-series images (40 optical sections) were acquired with a 0.75 NA 20× objective at a voxel size of 0.621-0.621-1.16 μm (x-y-z). For the analysis of colocalization of the Human Nuclei and GFAP, Z-series images (60 optical sections) were acquired with a 0.95 NA 40× objective at a voxel size of 0.3105-0.3105-0.58 μm (x-y-z). GFP-labeled cells were traced with Simple Neurite Tracer (Longair et al., 2011) in Fiji (Schindelin et al., 2012). Sholl analysis was performed with Simple Neurite Tracer with the cell body chosen as the center point. The data are presented as the mean ± standard error of the mean (SEM) from at least three independent experiments. Student’s *t* test, Tukey’s test or a repeated measure ANOVA test was performed. In all experiments, the samples and cells were analyzed in a double-blind manner.

## Results

**Figure 1:**
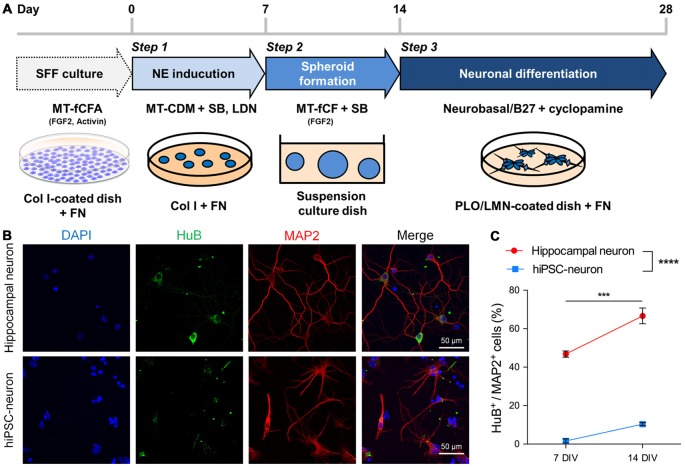
**Human induced pluripotent stem cells (hiPSC-neurons) on a glass dish barely expressed hippocampal neuronal markers. (A)** Schematic method for neuronal differentiation. The protocol consists of three steps: Step 1, NE induction (days 0–7) by dual-smad inhibition with SB and LDN; Step 2, spheroid formation (days 7–14) on suspension culture dishes in the presence of FGF2 and SB; Step 3, neuronal differentiation (days 14–28), by dissociating cells and plating them back on PLO/LMN-coated dishes in the presence of cyclopamine. SFF, single-cell and feeder-free; NE, neuroectoderm; SB, SB431542 (TGFβ inhibitor); LDN, LDN193189 (BMP inhibitor); Col I, collagen type I; FN, fibronectin; PLO, poly-L-ornithine; LMN, laminin. **(B)** Representative images of cultured hippocampal neurons (top) and hiPSC-neurons (bottom) at 14 days *in vitro* (DIV), immunostained with the hippocampal CA3 pyramidal cell marker HuB and the mature dendritic marker microtubule-associated protein 2 (MAP2). **(C)** The percentage of HuB^+^ cells/MAP2^+^ cells was 46.8 ± 1.44% at 7 DIV and 66.6 ± 3.64% at 14 DIV in hippocampal neurons and was 1.61 ± 0.91% at 7 DIV and 10.4 ± 0.71% at 14 DIV in hiPSC-neurons. Data are presented as the mean ± standard error of the mean (SEM). ***P < 0.001; hippocampal neuron 7 DIV vs. 14 DIV, **** *P* < 0.0001; hippocampal neuron vs. hiPSC-neurons both at 7 and 14 DIV, Tukey’s test after ANOVA, *N* = 5 samples for hippocampal neuron, four samples for hiPSC-neurons (each sample includes 42–114 cells).

We differentiated hiPSCs maintained with SFF culture (Ono et al., 2014) into cortical progenitor-like cells called hiPSC-neurons in the following three steps: NE induction, spheloid formation and neuronal differentiation (Figure 1A). These hiPSC-neurons were cryopreserved at the end of step 3. Cryopreserved hiPSC-neurons were thawed and cultured until immunocytochemistry was performed at 7 or 14 DIV. For comparison, we also cultured hippocampal neurons prepared from P1 mice. We found that almost half of hippocampal neurons that expressed the mature dendritic marker microtubule-associated protein 2 (MAP2) also expressed HuB, a marker for hippocampal CA3 pyramidal cells at 7 DIV, and the expression of HuB increased at 14 DIV (Figures 1B,C). In contrast, MAP2^+^ hiPSC-neurons scarcely expressed HuB at 7 DIV, and the expression was not significantly increased even at 14 DIV (Figures 1B,C). We also found that hiPSC-neurons possessed immature dendrites with shorter processes and fewer branches compared to cultured hippocampal neurons (Figure 1B).

**Figure 2:**
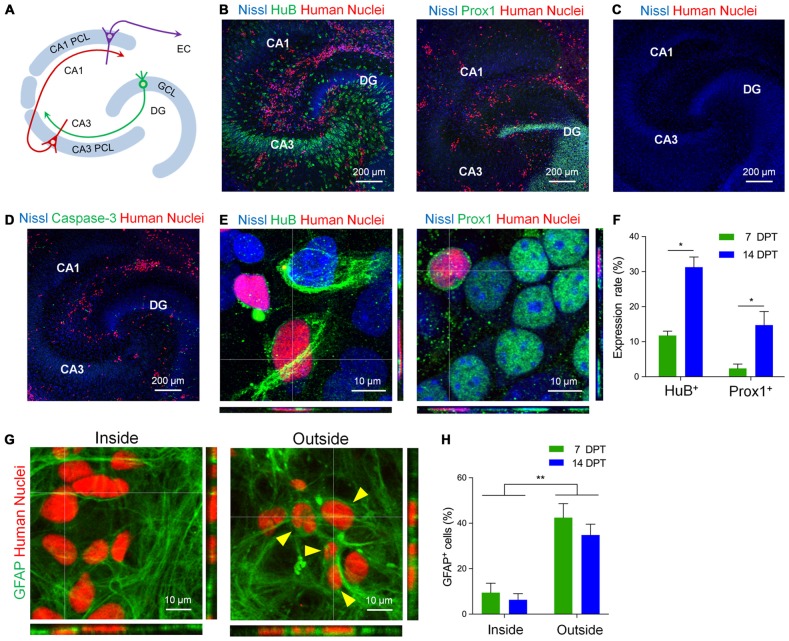
**hiPSC-neurons on hippocampal slice cultures expressed hippocampal neuronal markers. (A)** A schematic image of hippocampal architecture. There is a granule cell layer (GCL) in the dentate gyrus (DG) and a pyramidal cell layer (PCL). The PCL is divided into two types of layers, CA3 PCL and CA1 PCL, according to hippocampal subregions. Each cell layer contains crowds of cell bodies of neurons. EC, entorhinal cortex. **(B)** hiPSC-neurons (red, immunopositive for Human Nuclei) expressed the hippocampal CA3 pyramidal neuron marker HuB (left) and the DG granule neuron marker Prox1 (DG granule neuron marker; right) on cultured mouse hippocampal slices at 7 days post transplantation (DPT). **(C)** Cultured mouse hippocampal slices at 18 DIV that were not transplanted with hiPSC-neurons. **(D)** A representative image of cocultures immunostained with Human Nuclei and cleaved Caspase-3. hiPSC-neurons did not express cleaved Caspase-3. **(E)** Magnified images of hiPSC-neurons (red) that were immunopositive for HuB (left) and Prox1 (right) at 14 DPT. **(F)** The percentage of hiPSC-neurons that were immunopositive for HuB or Prox1 at 7 and 14 DPT. Data represent the mean ± SEM. * *P* < 0.05 vs. 7 DPT, Tukey’s test, *N* = 4–8 slices. **(G)** Magnified images of hiPSC-neurons immunopositive for Human Nuclei (red) and glial fibrillary acidic protein (GFAP; green) inside the CA1 PCL (left, Inside) and outside the neuronal cell layers (right, Outside). The hiPSC-neurons immunopositive for GFAP are indicated by arrowheads. **(H)** The percentage of hiPSC-neurons that were immunopositive for GFAP at 7 and 14 DPT. Data represent the mean ± SEM. ** *P* < 0.01, Tukey’s test after ANOVA, *N* = 5 slices each.

Next, to investigate whether hiPSC-neurons differentiate in pre-existing neural circuits, we transplanted hiPSC-neurons to mouse hippocampal slice cultures. Hippocampal slices (400 μm thick) were prepared from P6 mice and cultured as previously described (Koyama et al., 2007; Kasahara et al., 2016; Figure 2A). At 4 DIV, cryopreserved hiPSC-neurons were thawed and dropped onto cultured hippocampal slices. First, to examine how many cells were transplanted, we measured the density of hiPSC-neurons both inside and outside the neuronal cell layers in cultured slices. The density of transplanted hiPSC-neurons in the PCL was 3.19 ± 0.72 cells/100 μm^2^ (*n* = 7~35 cells/slice) at 7 days post transplantation (DPT) and 2.49 ± 0.39 cells/100 μm^2^ (*n* = 9~29 cells/slice) at 14 DPT. Outside neuronal cell layers, the density of transplanted hiPSC-neurons was 7.50 ± 0.72 cells/100 μm^2^ (*n* = 55~102 cells/slice) at 7 DPT and was 6.19 ± 0.95 cells/ 100 μm^2^ (*n* = 44~102 cells/slice) at 14 DPT. These results suggest that there is region-dependent difference in the density of engrafted hiPSC-neurons.

Next, we examined whether transplanted hiPSC-neurons express neuronal subtype-specific proteins such as the CA3 pyramidal cell marker HuB and the dentate granule cell marker Prox1 (Figure 2). At 7 DPT, we found a number of transplanted hiPSC-neurons that were immunopositive for Human Nuclei (Figure 2B), an antigen associated with the nuclei of human cells (Chen et al., 2016). We also immunostained the cultured slices that were not transplanted with hiPSC-neurons with anti-Human Nuclei, detecting no signals of Human Nuclei (Figure 2C). We found that 11.7 ± 1.1% (*n* = 10~165 cells/slice) of hiPSC-neurons in the CA3 PCL expressed HuB at 7 DPT and the expression increased to 31.3 ± 2.9% (*n* = 15~77 cells/slice) at 14 DPT (Figures 2E,F). In addition, we found that 2.3 ± 1.0% (*n* = 60~138 cells/slice) of hiPSC-neurons in the dentate GCL expressed Prox1 at 7 DPT, and the expression increased to 14.7 ± 3.3% (*n* = 12~49 cells/slice) at 14 DPT (Figures 2E,F). In this experimental system, we confirmed that transplanted hiPSC-neurons did not express the apoptotic marker Cleaved Caspase-3 at 7 DPT (Figure 2D), suggesting that hiPSC-neurons transplanted on cultured mouse hippocampal slices survive no less than 14 days.

We further examined whether hiPSC-neurons differentiate into non-neuronal lineages when transplanted in non-neuronal regions in the slice cultures, we examined the expression of the glial cell marker GFAP in transplanted hiPSC-neurons (Figure 2G). We found that 9.5 ± 3.7% at 7 DPT and 6.2 ± 2.4% at 14 DPT of hiPSC-neurons in the PCL expressed GFAP (Figure 2H). In contrast, outside the neuronal cell layers, 42.5 ± 5.5% at 7 DPT and 34.8 ± 4.3% at 14 DPT of hiPSC-neurons expressed GFAP (Figure 2H). These results raise the possibility that the factors from extracellular milieu in the neuronal cell layers are important for the neuronal differentiation of hiPSC-neurons.

**Figure 3:**
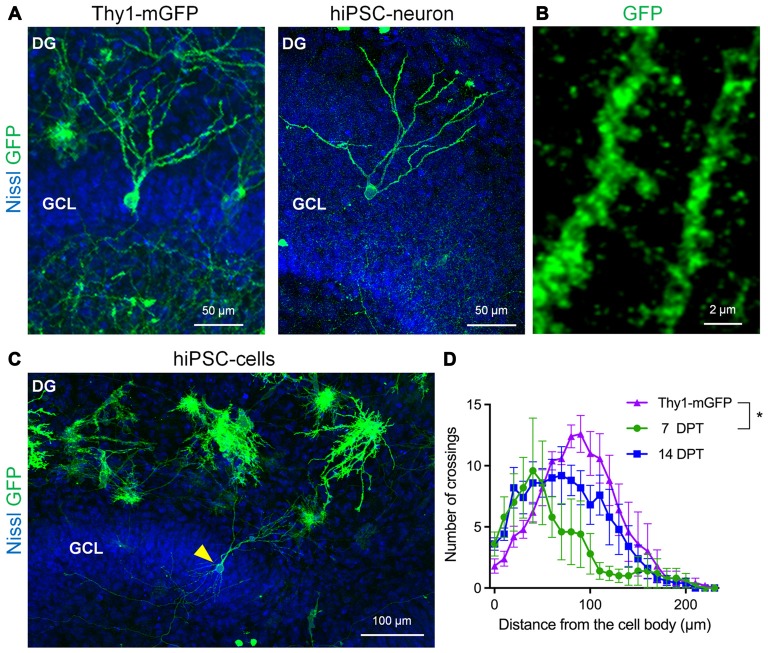
**hiPSC-neurons transplanted on the dentate GCL exhibited granule cell-like dendrites. (A)** Representative images of a Thy1-mGFP^+^ dentate granule cell in the GCL in the DG at 11 DIV (left) and a GFP^+^ hiPSC-neuron in the GCL at 14 DPT (right). **(B)** The magnified image of granule cell-like hiPSC-neurons (**A**, right) revealed dendritic spines. **(C)** A representative low magnification image of the DG. A granule cell-like hiPSC-neuron in the GCL is indicated by an arrowhead. **(D)** Sholl analysis of Thy1-mGFP^+^ granule cells and hiPSC-neurons in the GCL. The peak of crossing numbers of hiPSC-neurons was 40 μm at 7 DPT and shifted to 70 μm at 14 DPT toward the 90 μm peak of Thy1-mGFP^+^ granule cells. Data are presented as the mean ± SEM. * *P* < 0.05, repeated measures ANOVA. *N* = 5 cells for each group.

Next, we investigated whether transplanted hiPSC-neurons morphologically differentiate in cultured slices because neuronal morphology reflects the neuronal subtype-specific role and function in a defined neural circuit. To visualize the morphology of hiPSC-neurons, we electroporetically transfected hiPSC-neurons with mGFP before transplantation. For comparison, we also prepared slice cultures from Thy1-mGFP (Lsi1) mouse line (Figure 3A, left), in which mGFP expression is confirmed in the principal neurons in the hippocampus (Tao et al., 2016). At 11 DIV, Thy1-mGFP^+^ granule cells with polarized besom-shaped dendrites were found in the GCL of a Thy1-mGFP mouse slice culture (Figure 3A, left). Similarly, mGFP^+^ hiPSC-neurons that were transplanted in the GCL possessed granule cell-like dendrites (Figures 3A,C) with spine-like protrusions (Figure 3B) at 14 DPT. We quantitatively analyzed the similarity of dendritic morphologies between Thy1-mGFP^+^ granule cells and hiPSC-neurons in the GCL via Sholl analysis and found that from 7 to 14 DPT, the dendrites of hiPSC-neurons grew to resemble those of 11 DIV Thy1-mGFP^+^ granule cells (Figure 3C).

**Figure 4:**
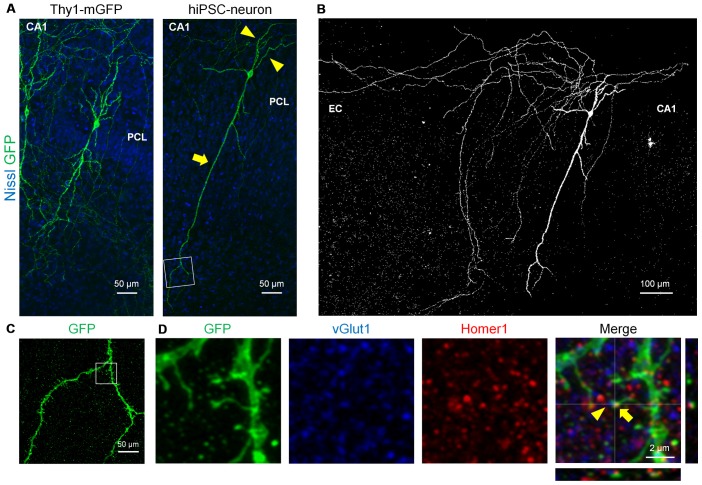
**hiPSC-neurons transplanted in the PCL exhibited pyramidal cell-like dendrites with synaptic spines. (A)** Representative images of a Thy1-mGFP^+^ pyramidal cell in the CA1 PCL at 11 DIV (left) and a GFP^+^ hiPSC-neuron in the PCL at 7 DPT (right). **(B)** A whole image of the GFP^+^ hiPSC-neuron in **(A)**. The GFP^+^ hiPSC-neuron projected its axons to the EC. **(C)** A magnified image of the boxed square in (**A**, right) revealed multiple spines along the dendrite. **(D)** Representative immunohistochemical images of the boxed square in **(C)**. The spine of the GFP^+^ hiPSC-neuron (arrows) was colocalized with the postsynaptic marker Homer1 and the presynaptic marker vGlut1 (arrowheads).

Finally, we examined whether transplanted hiPSC-neurons form spines and synapses, both of which are neuron-specific structures that are essential for synaptic transmission (Nimchinsky et al., 2001; Figure 4). At 7 DPT, mGFP^+^ hiPSC-neurons in the CA1 PCL exhibited a long apical dendrite (arrows) and multiple basal dendrites (arrowheads; Figure 4A, right), which resemble the dendritic morphology of Thy1-mGFP^+^ CA1 pyramidal cells (Figure 4A, left). Further, CA1 hiPSC-neurons possessed thin axon-like neurites that projected to the EC, mimicking the projection pattern of CA1 pyramidal axons *in vivo* (Amaral and Witter, 1989; Figure 4B). Magnified images of the dendrites of a CA1 hiPSC-neuron revealed a number of spine-like protrusions (Figure 4C), and these protrusions expressed the postsynaptic marker Homer1 and localized closely to the presynaptic marker vGlut1 (Figure 4D). These results suggest that hiPSC-neurons exhibited synapse-like structures.

## Discussion

In the present study, we differentiated hiPSCs into hiPSC-neurons and cultured them on glass dishes or mouse hippocampal slices. With our differentiation protocol (Figure 1A), human iPSCs differentiated into cortical progenitor-like cells, and approximately 10% of them expressed the hippocampal CA3 marker HuB at 14 DIV on glass dishes (Figure 1C). Interestingly, when cultured on mouse hippocampal slices, hiPSC-neurons localized on the CA3 PCL or the dentate GCL and expressed those neuron-specific markers (Figure 2F). However, hiPSC-neurons that did not express hippocampal neuron-specific markers at 14 DPT were present. These hiPSC-neurons may grow to exhibit hippocampal neuron-specific markers when cultured longer than 14 DPT. It is also possible that these hiPSC-neurons did not receive synaptic connections from pre-existing neural circuits, which are necessary for differentiation. The cellular and molecular mechanisms how hippocampal neuron-specific markers were expressed in transplanted hiPSC-neurons remain to be clarified. It is also possible that transplanted hiPSC-neurons that acquired properties of mature neurons were relatively viable: the majority of transplanted cells might die before 14 DPT while HuB- or Prox1-expressing cells survived.

The present study indicates that hiPSC-neurons can differentiate to gain characteristic neuronal properties and adapt to the surrounding environment in the mouse hippocampal slice cultures, though several studies have utilized ESCs and reported similar results. Mouse ESC-derived neural precursors were able to integrate into cultured mouse hippocampal slices in a region-specific manner (Neuser et al., 2013). Mouse ESC-derived neural precursors exhibited pyramidal or granule cell-like morphologies, formed dendritic spines and received synaptic inputs from surrounding neurons, although it was not examined whether mouse ESC-derived neural precursors expressed postsynaptic markers or hippocampal neuronal subtype-specific markers. In addition, human ESC-derived lt-NES (long-term self-renewing neuroepithelial stem) cells were delivered into the living mouse hippocampus (Steinbeck et al., 2012). Human ESC-derived lt-NES cells exhibited endogenous axonal projections, although they rarely expressed hippocampal neuronal markers. In the present study, we found that human iPSC derived-neurons localized on the CA1 PCL or the dentate GCL and exhibited pyramidal or granule cell-like morphology, respectively (Figures 3A, 4A). The dendritic morphology of granule cell-like hiPSC-neurons evolved with the duration of culture, resembling that of Thy1-mGFP^+^ granule cells (Figure 3D). The arborization of granule cell dendrites is performed by a transcription factor Prox1 (Iwano et al., 2012). In our co-culture system, Prox1 expression was induced in hiPSC-neurons on the GCL (Figure 2). Thus, it is likely that Prox1 played a role in the differentiation of hiPSC-neurons into cells with granule cell-like morphologies. However, the mechanisms of how Prox1 expression is induced in a region-specific manner remain to be answered. It is also important to note that the position of neurons itself is essential in shaping their dendritic arborization, likely in relation to region-specific afferent axonal innervations that synapse on their dendrites (Li et al., 2013). In the cortex of the developing mouse brain, the position of neurons determines cell fate and neuronal property, including dendritic morphology and the axonal projection pattern (Oishi et al., 2016).

There are several studies in which hiPSC-neurons were transplanted *in vivo* (Hargus et al., 2010; Tornero et al., 2013). However, it sometimes takes a couple of months to evaluate the integration of hiPSC-neurons *in vivo* (Nicholas et al., 2013), while it takes 2 weeks to evaluate the integration of hiPSC-neurons in our *ex vivo* culture system. Moreover, our *ex vivo* culture system will be suitable for time-lapse imaging of the integration of hiPSC-neurons and also for pharmacological experiments that enable the screening of factors required for efficient integration of transplanted hiPSC-neurons.

We also found that the pyramidal cell-like hiPSC-neurons in CA1 exhibited spine-like structures that were immunopositive for synaptic markers, indicating that the integrated hiPSC-neurons develop synaptic structures (Figures 4C,D). However, it should be noted that future studies using electrophysiological approaches and electron microscopy are necessary to confirm the presence of synapses on transplanted hiPSC-neurons. The pyramidal cell-like hiPSC-neurons also preserved the axonal projection pattern of the CA1 pyramidal neurons (Figure 4B). A previous study has shown that human ESC-derived medial ganglionic eminence (MGE)-like progenitors require as long as 7 months, which mimics the human neural development period, to develop into GABAergic interneurons with mature physiological properties (Nicholas et al., 2013). In our co-culture system, unexpectedly, hiPSC-neurons exhibited morphologically mature dendritic processes with synaptic protrusions within 2 weeks.

One of the challenges to be overcome in our co-culture system is the low efficiency of GFP-labeling of hiPSC-neurons. We transfected hiPSC-neurons with mGFP plasmids using *in vitro* electroporation and the transfection rate was as low as about 5%. Mainly because of this transfection issue, we could find a small number of GFP-positive of hiPSC-neurons in the neuronal cell layers of cultured slices. In future experiments, for example, the development and use of hiPSC-neurons in which GFP is genomically encoded may help solving the transfection issues.

It remains unclear how hiPSC-neurons were integrated into mouse hippocampal slices. In the subgranular zone of the hippocampus of the adult mouse brain, new granule cells are produced throughout life and are integrated into pre-existing neural circuits (Zhao et al., 2008). It has been reported that the neurotransmitter GABA from pre-existing inhibitory neurons is essential for the establishment of synaptic inputs on these newborn granule cells (Ge et al., 2006). This adult neurogenesis is enhanced by environmental enrichment, in which parvalbumin^+^ interneurons are known to play an important role (Alvarez et al., 2016). Thus, in the hippocampus, it is possible that GABAergic neurons are the key regulators that promote the integration of transplanted hiPSC-neurons into pre-existing neural circuits. Additionally, another study reported that the synaptic integration of newborn granule cells is controlled by astrocytes (Sultan et al., 2015). Because plenty of astrocytes exist in cultured hippocampal slices (Kasahara et al., 2016), it is likely that astrocytes also play a role in the incorporation of transplanted hiPSC-neurons, for example, by secreting proteins such as Thrombospondins to induce synapse formation (Christopherson et al., 2005). Another possibility is the cell fusion of hiPSC-neurons with existing hippocampal neurons in cultured slices. A previous study reported the fusion events of transplanted bone marrow stem cells with Purkinje cells in the recipients’ cerebellum (Alvarez-Dolado et al., 2003). Thus, we do not exclude the possible involvement of cell fusion events between hiPSC-neurons with hippocampal neurons, leading to the differentiation of transplanted hiPSC-neurons into hippocampal neuron-like cells.

In the present study, we developed a novel co-culture system of human iPSC-neurons and mouse hippocampal slices and showed that hiPSC-neurons are morphologically integrated into pre-existing neural circuits. In future studies, it will be necessary to investigate whether hiPSC-neurons can also differentiate functionally *in vivo*. Finally, our co-culture system has the potential to screen factors that are necessary for the efficient integration of transplanted iPSCs in the brain.

## Author Contributions

TH conducted and analyzed the experiments and wrote the manuscript. MA prepared primary neuronal cultures. TA, TS, and TO prepared hiPSC-neurons. RK designed and planned the project and wrote the manuscript. YI discussed the results and commented on the manuscript.

## Funding

This work was supported by Japan Society for the Promotion of Science (JSPS) KAKENHI Grant Numbers 26460094 and 26117504.

## Conflict of Interest Statement

The authors declare that the research was conducted in the absence of any commercial or financial relationships that could be construed as a potential conflict of interest.
